# Application of Stereo Digital Image Correlation on Facial Expressions Sensing

**DOI:** 10.3390/s24082450

**Published:** 2024-04-11

**Authors:** Xuanshi Cheng, Shibin Wang, Huixin Wei, Xin Sun, Lipan Xin, Linan Li, Chuanwei Li, Zhiyong Wang

**Affiliations:** 1 School of Mechanical Engineering, Tianjin University, Tianjin 300350, China; cxstj@tju.edu.cn (X.C.);; 2 School of Civil Engineering and Architecture, Nanchang University, Nanchang 330000, China

**Keywords:** facial expressions, digital image correlation, deformation, dynamic analysis

## Abstract

Facial expression is an important way to reflect human emotions and it represents a dynamic deformation process. Analyzing facial movements is an effective means of understanding expressions. However, there is currently a lack of methods capable of analyzing the dynamic details of full-field deformation in expressions. In this paper, in order to enable effective dynamic analysis of expressions, a classic optical measuring method called stereo digital image correlation (stereo-DIC or 3D-DIC) is employed to analyze the deformation fields of facial expressions. The forming processes of six basic facial expressions of certain experimental subjects are analyzed through the displacement and strain fields calculated by 3D-DIC. The displacement fields of each expression exhibit strong consistency with the action units (AUs) defined by the classical Facial Action Coding System (FACS). Moreover, it is shown that the gradient of the displacement, i.e., the strain fields, offers special advantages in characterizing facial expressions due to their localized nature, effectively sensing the nuanced dynamics of facial movements. By processing extensive data, this study demonstrates two featured regions in six basic expressions, one where deformation begins and the other where deformation is most severe. Based on these two regions, the temporal evolutions of the six basic expressions are discussed. The presented investigations demonstrate the superior performance of 3D-DIC in the quantitative analysis of facial expressions. The proposed analytical strategy might have potential value in objectively characterizing human expressions based on quantitative measurement.

## 1. Introduction

As the most direct way to communicate emotions, facial expression is first noted by Darwin [1]. The research on facial expressions has been applied to lie detection, human–computer interaction, human-face robots, etc. [2,3]. Basically, there are six acknowledged basic emotions, including happiness, surprise, sadness, fear, disgust and anger [4,5]. Correspondingly, there are six basic facial movements that act as expressions to reflect emotions. In past decades, research on facial expressions reveals more details. Not only were more facial expressions such as contempt [6], shame [7], pride [8] and guilt [9] studied, but an additional 15 compound facial expressions were discovered and defined [10,11]. Even though human beings have very rich expressions and facial movements under different expressions are very complex, humans can always distinguish and understand the expressions of others through visual information from their faces [12]. Therefore, in order to reveal how humans recognize expressions through facial deformation movements and understand the underlying mechanisms of expressions, many tools for measuring and analyzing expressions have been proposed [13]. However, these methods inherently have limitations in objective quantitative measurements and full-field analysis, highlighting a significant research gap. This creates a serious problem: the absence of thorough dynamic analysis for the quantitative assessment of expressions. For example, as the most commonly used method in expression analysis, the Facial Action Coding System (FACS) [14,15,16] allows for individual encoding of each expression. However, this process is conducted through visual observation and lacks quantification. While facial electromyography (EMG) is a sensitive and objective measurement method, it requires the placement of electrodes on the participant’s face in a specific configuration. An individual typically can tolerate the placement of only a few electrodes on their face at one time, which considerably limits its application. Currently, there is also scant literature providing related research [13].

This paper is motivated by the need for a comprehensive understanding of facial expressions and introduces a full-field, high-precision analysis of three-dimensional deformation fields of facial movements. Such an approach marks a significant contribution to the field of facial expression analysis by exploring the potential of 3D-DIC in the dynamic analysis of facial expressions. For specializing 3D-DIC in expression analysis, some improvements on it are proposed. The specialized 3D-DIC is applied to analyze the forming processes of 6 basic expressions with a time resolution of 0.04 s, sensing the nuanced changes over time. The dynamic 3D-DIC measurement provides us with a wealth of data available. From the huge available data measured by 3D-DIC, the crucial datasets for facial expressions are identified. The obtained displacement fields demonstrate a good agreement with the developed FACS. Furthermore, the strain field has the ability to highlight the local tension or contraction of the muscles involved in each expression, effectively sensing the underlying muscular activities. Building on the measured dynamic process of different expressions, two featured regions are proposed to characterize their temporal evolutions. The experimental results demonstrate the deformation processes of the six basic facial expressions and show the potential of 3D-DIC in objectively identifying the facial expressions.

## 2. Literature Review

In the first place, to standardize the process of recognizing facial expressions, the FACS is proposed to encode the movements of individual facial muscles, thereby characterizing different emotional expressions and analyzing the small discrepancy in facial expressions. It has become the most commonly used tool for analyzing expressions, offering a comprehensive framework for researchers to systematically study the nuances of human emotions. Building on this foundation, recent studies in facial expression analysis have utilized a range of analytical methods and tools to understand the complexities and variabilities in emotion perception and expression. Among these, in recent works, a hybrid Bayesian network model has been employed to analyze dependencies between facial action units (AUs) within FACS, thereby enhancing AU recognition [17]. Furthermore, the method that combines the FACS with statistical analysis has been employed for cross-cultural studies of expressions [18,19]. But, the use of FACS requires plenty of time for preliminary training and analysis, and the results might have subjective bias depending on the users [20,21]. Especially in dynamic analysis, the FACS needs to process each frame, which is very time-consuming. In the cognitive neuroscience domain, neuroimaging techniques are used to demonstrate the significance of visual exposure to faces in the development of specialized cortical areas for face recognition [22]. Additionally, mathematical modeling and classification techniques have been used to investigate the multifunctionality of smiles within social contexts [23]. Expressions are dynamic processes, yet these methods still do not fully capture the analysis of dynamic details.

Moreover, as a computer vision technique used to estimate the motion of objects or image features across a sequence of frames, the optical flow [18] has been widely used in the automated analysis of human expressions [24]. But, the different optical flow methods have consistently faced some common challenges from the complexity of the facial deformation during various expressions [25,26]. Therefore, as a type of rapid and abrupt facial movement, there is still a lack of objective methods capable of efficiently conducting dynamic analysis of expressions at a high temporal resolution and uncovering their full-field detail information.

DIC is another developed optical method for deformation measurements. It can provide simultaneous displacement and strain fields with high accuracy. Furthermore, its three-dimensional version, 3D-DIC, is able to obtain the three-dimensional displacements of none-plane subjects through stereovision techniques [27,28]. Given the advantages like high accuracy, global field, non-contact, etc., DIC has been widely available to many scientific and engineering fields [29,30,31]. Recently, DIC has increasingly become one of the important measuring methods of living human skin. Staloff et al. [32,33] measured the deformation of the skin surface by two-dimensional DIC (2D-DIC). Nagisa et al. [34,35] proposed a method to calculate the strain from three-dimensional displacements and measured the strain fields around the human eye during the blink by 3D-DIC. Cheng et al. [36] proposed the SF-DIC on biological skin to reduce the impact of artificial speckles. However, there is currently a scarcity of detailed research on the application of DIC in the analysis of facial expressions.

## 3. Method

In this section, we initially provide a concise overview of 3D digital image correlation (3D-DIC) to ensure the completeness of our work. For a more comprehensive understanding of this topic, readers can refer to related review works [28]. Furthermore, to adapt the 3D-DIC to the deformation measurement of facial expression, we customized some key improvements to ensure the experimental measurement with high resolution, low noise level and acceptable computing loading. After that, a novel analysis method based on the strain field from 3D-DIC is proposed to further reveal the complexity of expressions.

### 3.1. The Principle of 3D-DIC

**Figure 1:**
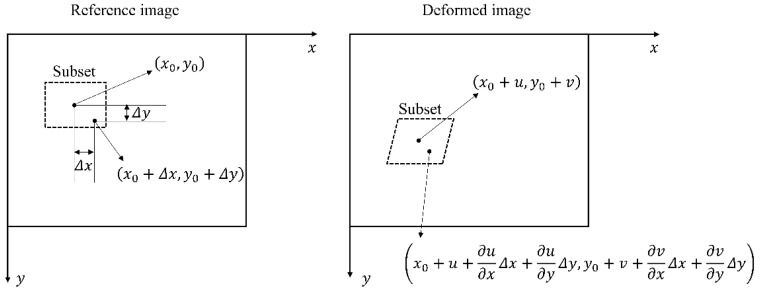
The principle of 2D-DIC.

The 3D-DIC is based on the 2D-DIC and the stereovision technique. The 2D-DIC is an optical method for 2D deformation measurement by the images from one single camera. Assuming F(x,y) represents the gray level at position (x,y) in the image of object before deformation and $G(x^{\prime },y^{\prime })$ represents the gray level at position $\left(x^{\prime },y^{\prime }\right)$ in the image of object after deformation. In addition, the first-order shape function is used. As shown in Figure 1, the displaced position in certain subsets can be obtained as follows:

(1) $$\begin{array}{l} x^{\prime }=x_{0}+u+\frac{\partial u}{\partial x}\Delta x+\frac{\partial u}{\partial y}\Delta y\\ y^{\prime }=y_{0}+v+\frac{\partial v}{\partial x}\Delta x+\frac{\partial v}{\partial y}\Delta y \end{array}$$

where u and v represent the displacements of the central position $\left(x_{0},y_{0}\right)$ at horizontal and vertical direction, respectively, and $\frac{\partial u}{\partial x}$, $\frac{\partial u}{\partial y}$, $\frac{\partial v}{\partial x}$, $\frac{\partial v}{\partial y}$ are the gradients of the displacement.

The displacements parameters u, v, $\frac{\partial u}{\partial x}$, $\frac{\partial u}{\partial y}$, $\frac{\partial v}{\partial x}$, $\frac{\partial v}{\partial y}$ can be obtained by minimize the following correlation coefficient:

(2) $$C=-\frac{\sum_{x=1}^{2M+1}\sum_{y=1}^{2M+1}\left[F\left(x,y\right)-F_{m}\right]\times \left[G\left(x^{\prime },y^{\prime }\right)-G_{m}\right]}{\sqrt{\sum_{x=1}^{2M+1}\sum_{y=1}^{2M+1}{\left[F\left(x,y\right)-F_{m}\right]}^{2}}\times \sqrt{\sum_{x=1}^{2M+1}\sum_{y=1}^{2M+1}{\left[G\left(x^{\prime },y^{\prime }\right)-G_{m}\right]}^{2}}}$$

where the 2*M*+1 is the length of the subset, $F_{m}$ and $G_{m}$ represent the grayscale mean values of the subset of the reference image and deformed image, respectively.

**Figure 2:**
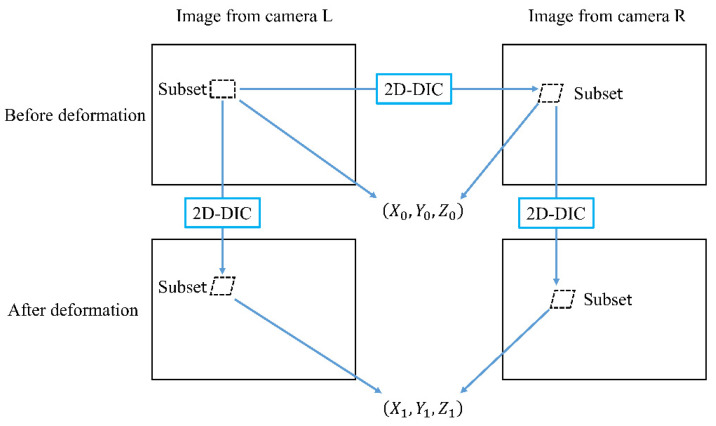
The outline of 3D-DIC

As shown in Figure 2, 3D-DIC is able to obtain the 3D deformation data based on a pair of 2D-DIC analyses using the images from two cameras located at different angles. The coordinates $\left(X_{0},Y_{0},Z_{0}\right)$ and $\left(X_{1},Y_{1},Z_{1}\right)$ are the three-dimensional coordinates of the central point of the subset on the image before and after deformation, respectively. The corresponding position on the image from camera R or the image after deformation can be obtained from the algorithm introduced before. The corresponding positions in the image from camera L and camera R are able to determine the three-dimensional coordinates through the stereovision technique [37]. Then, the three-dimensional displacements can be acquired from the three-dimensional coordinates before and after deformation.

### 3.2. Customized 3D-DIC for Facial Deformation Analysis

To make 3D-DIC suitable for the measurement of facial deformation during expressions, we propose the following customization.

Automatic partial calculation: In the computation of DIC, speckle patterns are one of the most important factors. The speckle provides randomness and enables every subset to have unique gray information. However, to capture more detailed deformations of the face, high-resolution cameras might be utilized. High-resolution images typically necessitate larger subset sizes, which consequently lead to an increase in computational load, proportional to the square of the subset size. In fact, in a subset of high-resolution images, there are plenty of pixels with low gray information that reduce the accuracy of DIC analysis according to the previous research work, and the gray information of pixels can be determined by the sum of squared gradients of its gray values [38]. In this work, those pixels with gray information of the top 70% in a subset are used. This approach significantly enhances computational efficiency without compromising the accuracy of the calculations. Further, the irregularly selected pixels also have an additional effect on depressing the inevitable systematic error of DIC [39].

Self-adaptive reference image selection: DIC is fundamentally a method for acquiring deformation data from a pair of images, typically referred to as the reference image and the deformed image. The selection of these two images directly impacts the meaning and accuracy of the obtained data. Given the focus of this research on the dynamic analysis of facial expressions, the entire formation process will be examined. Therefore, it is essential to consistently use the first image as the reference image to capture the complete deformation process. However, a time resolution of 0.04 s in this dynamic research indicates dozens of images are required for calculation. Furthermore, the significant time gap between two images may lead to the direct calculation of large deformations, potentially compromising accuracy. To address this issue, during the nth calculation, if more than 10% of the points of interest (POIs) have coefficients larger than −0.9, the (n − 1)th image is designated as the reference image. Subsequently, the results of the nth calculation are adjusted by integrating the results from the (n − 1)th calculation. This process continues until the next deterioration in computational performance is observed, ensuring accuracy in the presence of large deformations.

### 3.3. Dynamic Deformation Analytical Strategy

**Figure 3:**
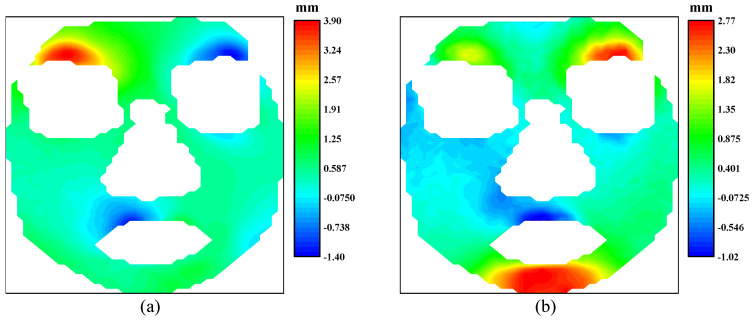
The displacement fields of the human face: (**a**) displacement field at horizontal direction; (**b**) displacement field at vertical direction.

**Figure 4:**
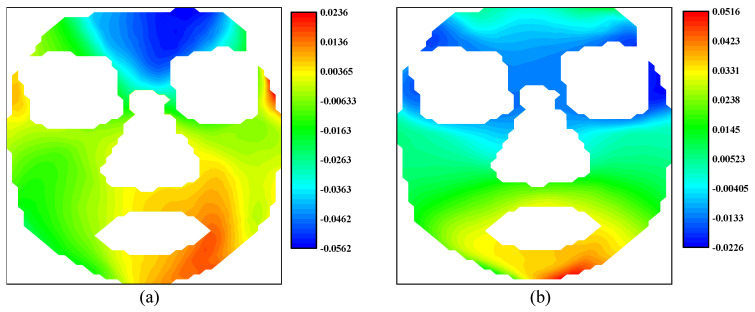
The strain fields of the human face: (**a**) horizontal strain field; (**b**) vertical strain field.

As illustrated before, the 3D-DIC is able to obtain the three-dimensional displacements on the surface of a none-plane subject. Figure 3 shows an example of facial displacement fields in the horizontal direction and vertical direction calculated by 3D-DIC. In a broad sense, displacement can be viewed as the integration of local strain along a specific path. From this perspective, displacement is a global quantity, while strain is a localized one. In this sense, we believe that strain has a more direct association with local muscle movements during facial expressions compared to displacement. Therefore, strain data should also be given careful consideration to analyze facial expressions. Nagise et al. [34] give a simple method to obtain the strain distribution on three-dimensional surfaces from the three-dimensional displacements. The strain fields at horizontal direction and vertical direction corresponding to Figure 3 are shown in Figure 4. Figure 3a presents obvious displacements around the eyebrows. Based on daily cognition, these obvious displacements are caused by the contraction near the middle of the eyebrows. Figure 4a illustrates the significant contraction strain in that region. Therefore, strain fields offer significant advantages in capturing and describing the local tension and contraction.

**Figure 5:**
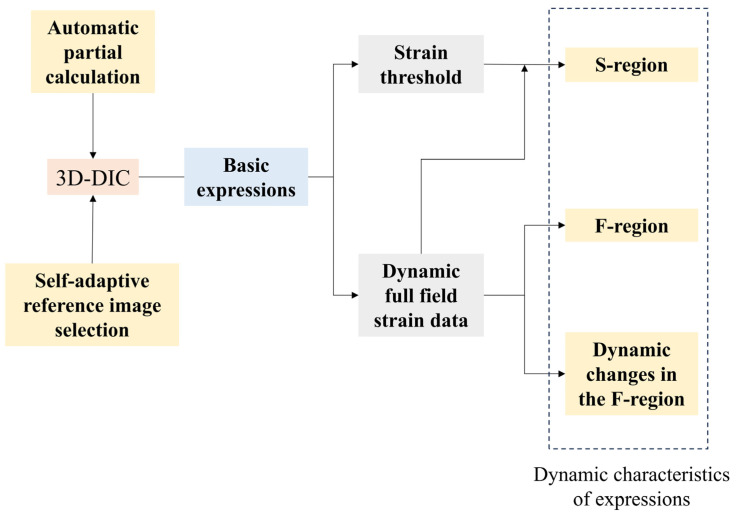
The implementation process of dynamic analysis.

In the research of facial expression analysis, there are two issues that often receive attention. One is where the facial movement of an expression starts up, and the other is where the facial movement fluctuates the most over time. As indicated by the results of the following experiments, two key regions identified through calculated strain are very useful for us to characterize the difference among the six basic expressions. They are named the starting-up region (S-region) and the maximum fluctuated region (F-region). The S-region can be determined by the area where strain first reaches a threshold. The F-region is the location where strain fluctuation is most apparent during the evolution of an expression. The more dynamic details of expressions could be obtained by tracking the strain changes in such regions. The implementation process of this work is shown in Figure 5.

## 4. Experiments and Results

In this section, the experimental setup is introduced first. Then, the threshold to judge if the expression occurs is proposed by a baseline experiment. Afterward, a detailed analysis of the forming processes for the six basic expressions is conducted, utilizing the measurement results obtained from 3D-DIC. Furthermore, some typical features of the basic expressions are identified based on their S-regions and F-regions.

### 4.1. Experimental Environment and Set-Up

**Figure 6:**
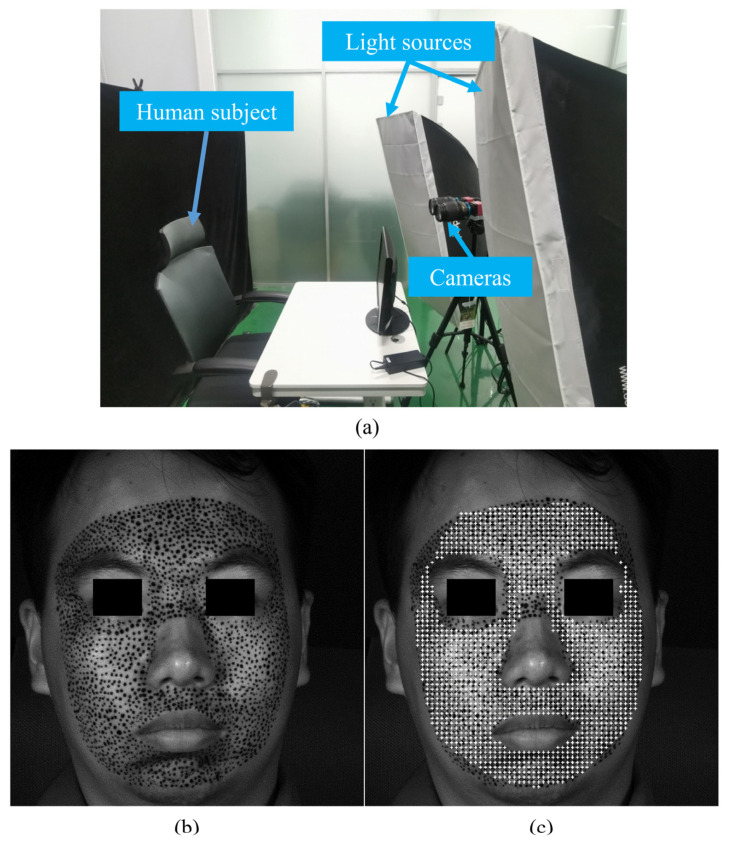
Experiments for human subject: (**a**) experimental set-up; (**b**) recorded original image; (**c**) the distribution of POIs.

The experimental setup is shown in Figure 6a. The setup includes two light sources and two 8-bit CMOS cameras produced by the South Korean company View Works (Gyeonggi-do, Republic of Korea). Since the high-resolution images enable a denser distribution of POIs, detailed information can consequently be analyzed more effectively. The image resolution in this work is 2560 × 2560 pixels. And a pixel corresponds to the actual about 0.08 mm. The strain at each point is derived from the displacement fit of all points within a 4.2 mm radius around it, representing the uniform strain of that region.

An example of recorded images is demonstrated in Figure 6b. The male subject is 25 years old and in good health. The artificial speckle was made on the face in advance. The influence of artificial speckles on the calculation has been given in previous research [40,41,42]. Figure 6c gives the distribution of POIs for calculation, and the white cross marks indicate the POIs. The space between POIs is 35 pixels in both a horizontal direction and a vertical direction. The total number of POIs is 1539.

The specific parameters and algorithms in DIC are set as follows. The subset size of each POI is 61 × 61 pixels. The Newton–Raphson iteration method with first-order shape function and cubic B-spline interpolation were employed in the DIC algorithm.

### 4.2. Experiment for Neutral Status

**Figure 7:**
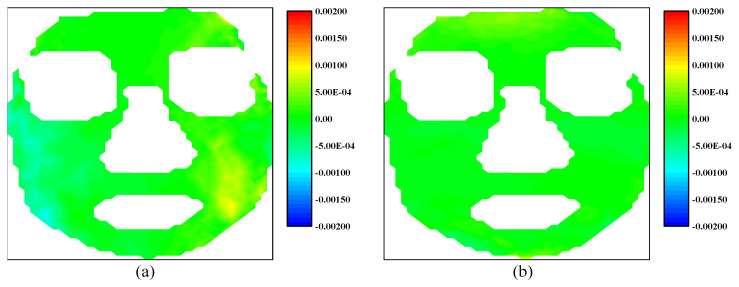
The strain fields of the human face in neutral status: (**a**) horizontal strain field; (**b**) vertical strain field.

Since the experimental subject is a living human, even though the experimental subject stays neutral, the strain caused by breath or other physiological activities exists on the face. Furthermore, the calculation error influences the experimental results as well. To confirm the range of strain of this part, a preliminary experiment was carried out. The subject is required to be static and neutral within a short time. A total of 10 images were captured with a time interval of 0.04 s. The first recorded image is used as the reference image, and the other 9 images are used as the deformed images. The strain fields of the subject in neutral status at a certain moment are shown in Figure 7, which displays the strain magnitude when the subject is under neutral status.

**Figure 8:**
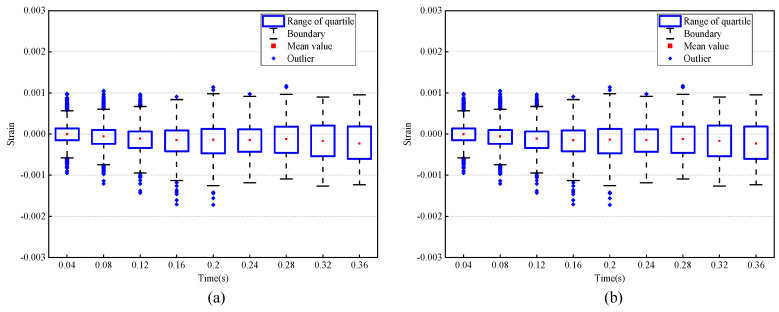
Statistical results of strain of neutral status: (**a**) horizontal strain statistical results; (**b**) vertical strain statistical results.

The statistical results of the horizontal and vertical strains in neutral are shown in Figure 8. The red point indicates the change in the mean value, the blue rectangular frame represents the range from lower quartile to upper quartile, the black dotted line is the boundary and the blue rhombus is an outlier. From Figure 8, it can be found that the strain in neutral is no more than 0.002 in both horizontal and vertical directions, based on the above results. Therefore, a threshold of 0.002 for the strain was used to determine whether the deformation in the subsequent experiments was caused by expressions.

### 4.3. The Temporal and Spatial Characteristics of the Six Basic Expressions

In this experiment, the forming processes of expressions of the subject are recorded. The 3D-DIC supplied the evolution history of the displacement and strain fields of each expression. Leveraging the extensive data acquired from experimental measurements, detailed full-field analyses of each expression were conducted to examine both spatial and temporal characteristics. To obtain the spatial characteristics of each expression, we present its typical displacement and strain fields and illustrate the consistency between the fields and the AUs of the classical FACS. It should be noted that the different investigations proposed the different compositions of AUs of each expression [13]. In this work, we only focus on those universal AUs of each expression in the different literature. In addition, the FACS is FACS is an intuitive judgment method based on visual observation. It involves the visual observation of specific muscle movements. Therefore, this paper only identifies the consistency between the FACS and the quantitative measurement results. After that, two regions, referred to as S-region and F-region, as previously mentioned, are defined and identified to characterize the temporal evolution of each expression.

According to Section 3.2, the POI whose strain magnitude reaches 0.002 first is regarded as the POI of starting up (S-POI). Additionally, in the forming process of a certain expression, the strain variance over time at each POI is considered as its fluctuation, and the 100 POIs with the most fluctuation are referred to as F-POIs. The following experimental results suggest that both S-POIs and F-POIs are clustering rather than being randomly distributed. Hereafter, these clusters are referred to as S-Region and F-Region, respectively. More specifically, the S-regions and F-regions derived from the horizontal and vertical strains exhibit distinct features of each expression. Thus, the horizontal/vertical S-region and F-region will be analyzed separately to show their unique features.

In the following, the measurement results and the deformation features of all six basic expressions will be presented. In order to display the specific locations of S-regions and F-regions on the face clearly and compare the differences of these regions between different expressions, we have marked the key points on facial images recorded before the expressions start at each experiment.

#### 4.3.1. Happiness

**Figure 9:**
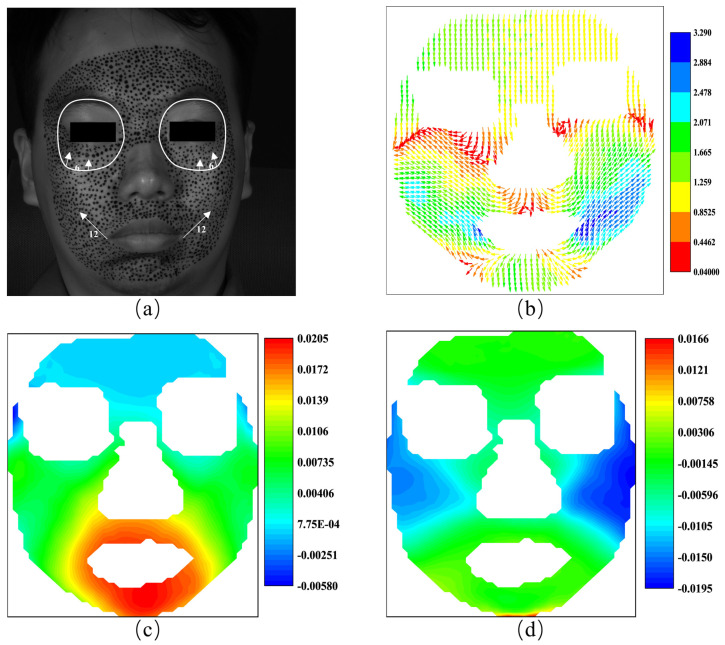
The AUs and the vector field and the strain fields at the expression of happiness: (**a**) the AUs caused by happiness; (**b**) the vector field of happiness; (**c**) horizontal strain field; (**d**) vertical strain field.

Figure 9a shows the AU 6 and AU 12 of FACS when the subject is expressing happiness. AU 6 is the cheek raiser caused by orbicularis oculi and pars orbitalis, while AU 12 is the lip corner puller by zygomatic major. In Figure 9b, we use the color of the arrows to represent the magnitude of the vectors, rather than their length, to alleviate the inconvenience caused by the invisibility of vectors with low magnitude. As shown in Figure 9b, the displacement vectors display an upward trend in the area below the eye sockets, consistent with the movement represented by AU6, although it is not perfectly symmetrical in specific directions. At the corners of both sides of the mouth, the displacement vectors are inclined upward, consistent with the movement represented by AU12, but the magnitude on the right side is noticeably greater than on the left side. For the results of this measurement, the movement of AU 12 is larger than that of AU 6. Furthermore, as shown in Figure 9b, the movement of AU 6 displays a weak asymmetry since the movement on the right side is larger than that on the left side.

Figure 9c,d shows the horizontal and vertical strain fields calculated from the displacement field in Figure 9b. As discussed previously, strain datum has advantages in characterizing expressions because they can provide localized information. For example, movement of the mouth corners will inevitably cause horizontal tension of the skin around the mouth. And the obvious horizontal tension, i.e., positive stain, is exactly the main feature of Figure 9c. As far as the vertical stain is concerned, although the movement of AU 6 and AU 12 are both upward, the movement magnitude of AU 12 is greater. Therefore, significant contraction is expected to occur on the cheek, a feature that is prominently highlighted in Figure 9d. It appears that strain fields can serve as a valuable alternative to displacement fields for characterizing this expression. The subtle variations observed in the horizontal and vertical strain fields reveal specific details in the facial expression. In Figure 9c, the positive strain around the mouth area indicates the activation of muscles associated with lip movement. This confirms the presence of happiness expression in the subject. Similarly, Figure 9d demonstrates negative strain in the cheek region, suggesting the involvement of muscles related to cheek movement or contraction. By examining these strain fields, we can gain insights into the dynamics and intensity of facial expression, contributing to a more detailed understanding of human emotional cues.

**Figure 10:**
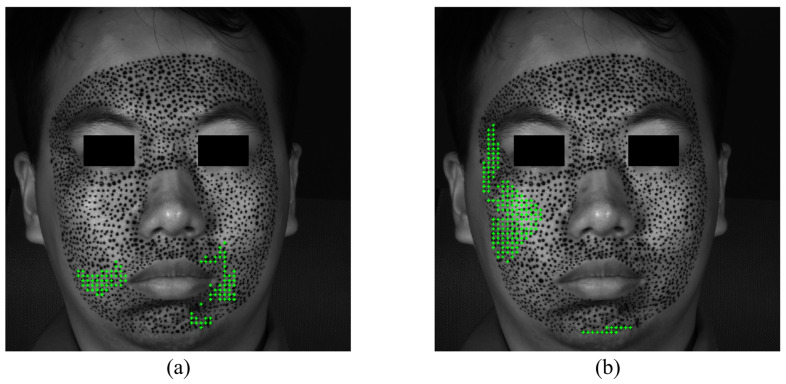
The S-POIs at the expression of happiness start: (**a**) horizontal S-POIs; (**b**) vertical S-POIs.

The S-POIs of happiness are shown in Figure 10. Although not very rigorous, in order to quantitatively analyze and compare the priming characteristics of different expressions, this paper supposes that facial expressions are considered to begin when there are at least 10 POIs exceeding the strain of 0.002. Figure 10a,b demonstrate the specific locations of S-POIs, i.e., the green cross marks, in the expression of happiness. From Figure 10a,b, it can be easily found that there are apparent S-regions in the expression of happiness because the S-POIs are concentrated in some locations, instead of randomly distributed. This indicates that such locations are first obviously deformed in the forming process of happiness. Both the horizontal and vertical S-regions display some asymmetry. This could provide more in-depth insight for us to describe the activation of an expression. With respect to the happiness of this experiment, the horizontal S-regions are located at the mouth corners, and the vertical S-region is mainly distributed at the left cheek.

**Figure 11:**
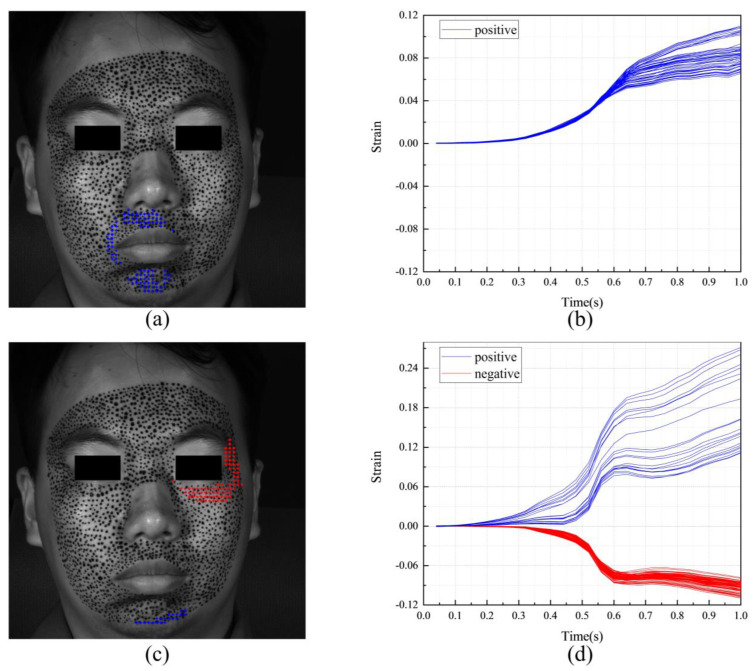
The F-region and the strain evolutions of F-POIs in the expression of happiness: (**a**) the horizontal F-region; (**b**) the horizontal strain evolutions of horizontal F-POIs; (**c**) the vertical F-region; (**d**) the vertical strain evolutions of vertical F-POIs.

Figure 11 shows the F-region and the strain evolutions of F-POIs under happiness. Figure 11a,c show the positions of the F-regions. In them, the blue color and the red color represent the F-POIs with positive (tension) and negative (contraction) stains, respectively. For this measurement, the F-regions are around the mouth and eye. It also should be noticed there are obvious differences between the S-region in Figure 10 and the F-regions in Figure 11a,b. This means that the region that is first activated is inconsistent with the region that undergoes the most intense tension or contraction. Figure 11b,d shows the strain evolutions of each F-POI. The strain evolution curves of the F-POIs are with similar shapes and different magnitudes. While the deformation progressively unfolds over the entire 1 s duration, it is most rapid and pronounced between 0.4 and 0.7 s.

#### 4.3.2. Anger

**Figure 12:**
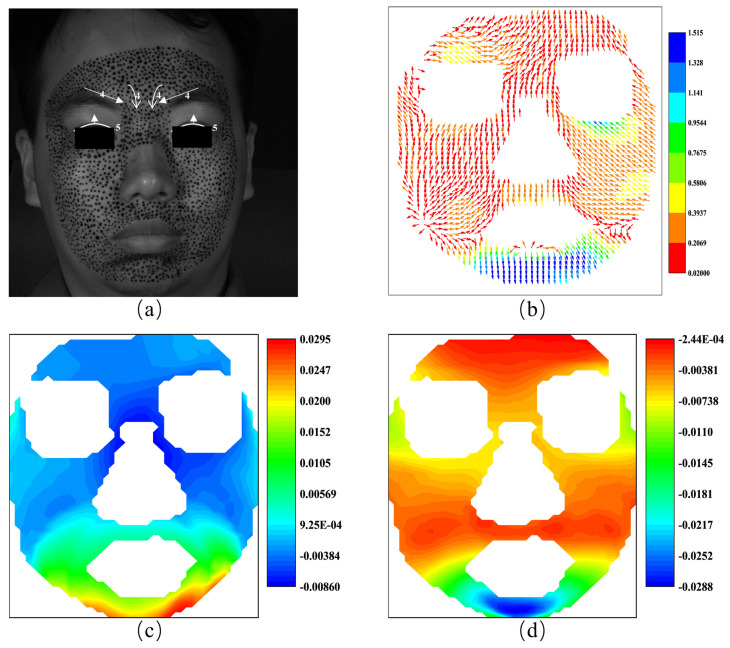
The AUs and the vector field and the strain fields at the expression of anger: (**a**) the AUs caused by anger; (**b**) the vector field of happiness; (**c**) horizontal strain field; (**d**) vertical strain field.

The AU compositions of the classic FACS, the displacement vector and the horizontal and vertical strain fields of the subject under anger are shown in Figure 12. Figure 12a shows the AU 4 and AU 5 of the anger. Due to safety considerations for the subject, no necessary speckle was made in the area of AU 5 during the experiment, so we will not discuss it here. AU 4 is characterized by the drawing together of the inner parts of the eyebrows and is mainly caused by the procerus. As shown in Figure 12b, the counterpart of AU 4 is evident on the left side, but the displacement vectors on the right side show some asymmetry. Figure 12b shows asymmetry in the activation of AU 4, reflecting the complex nuances of an angry expression.

Moreover, the strain fields depicted in Figure 12c,d clearly illustrate negative strain, i.e., contraction, around the glabella in both horizontal and vertical directions. This is a direct reflection of AU 4. The strain fields in Figure 12c,d reveal a pronounced contraction at the glabella, hinting at a connected muscular response that might extend to the forehead, suggesting a composite reaction rather than an isolated procerus action. This asymmetry might be the key to unscrambling the subtleties of individual facial expressions.

**Figure 13:**
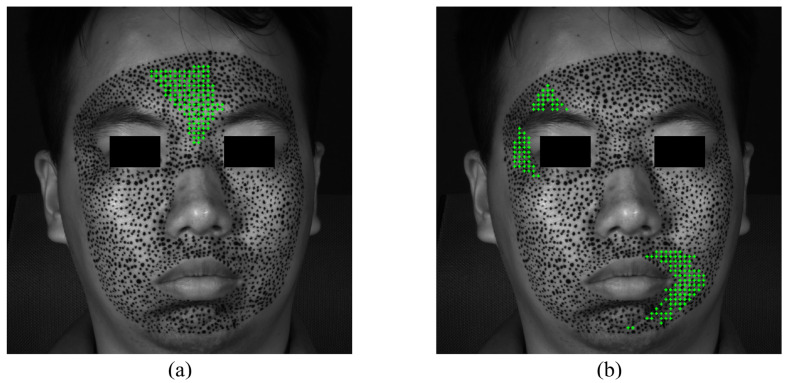
The S-POIs at the expression of anger: (**a**) horizontal S-POIs; (**b**) vertical S-POIs.

Figure 13 highlights the S-POIs for the anger expression, with the horizontal S-region between the eyebrows indicating initial frowning due to procerus contraction. The vertical S-region, marked by S-POIs around the eyes and mouth, shows the early skin tension during the anger display. These S-regions are pivotal in detecting the beginning of anger, revealing the primary muscle groups responsible for the facial deformation characteristic of this emotion.

**Figure 14:**
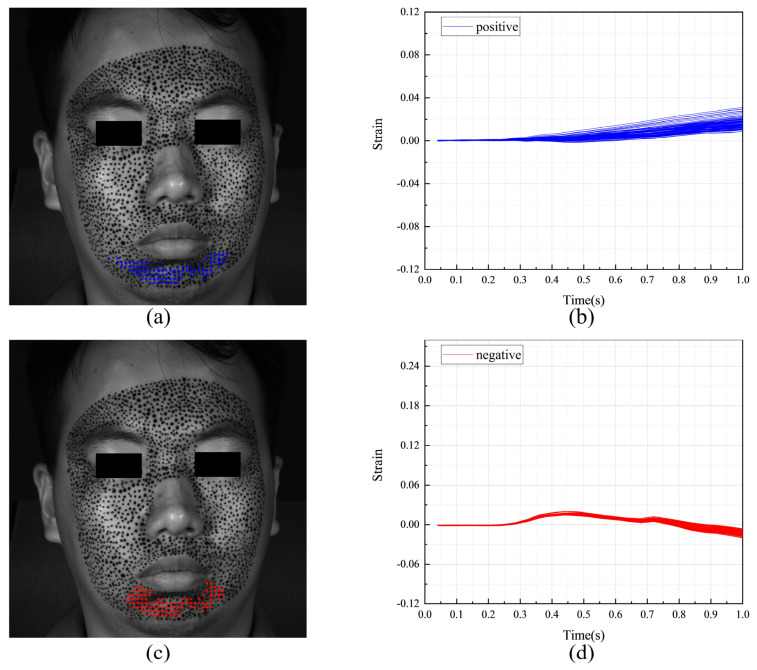
The F-region and the strain evolutions of F-POIs in the expression of anger: (**a**) the horizontal F-region; (**b**) the horizontal strain evolutions of horizontal F-POIs; (**c**) the vertical F-region; (**d**) the vertical strain evolutions of vertical F-POIs.

The F-region and the strain time evolutions of F-POIs are demonstrated in Figure 14. Figure 14a,b show the horizontal F-POIs and their strain evolutions, while Figure 14c,d demonstrate the vertical F-POIs and their strain evolutions. The horizontal F-region and the vertical F-region are both on the jaw and extend to the location close to the corner of the mouth. This indicates that the most active area of the expression of anger is near the jaw. Approximately, the horizontal strain in Figure 14b remains positive, while the vertical strain in Figure 14d stays negative. Compared to the result of the happiness experiment in Figure 11b,d, the strain evolutions of anger are less obvious, but the horizontal strain and vertical strain diverge at the same time, at about 0.3 s. Compared to the clear facial changes seen with happiness, the strain patterns for anger are more subtle, hinting at a less intense emotional response. The strains that occur together around 0.3 s might highlight a point where the expression of anger becomes fully visible on the face, which may correspond with the peak intensity of the anger emotion.

#### 4.3.3. Disgust

**Figure 15:**
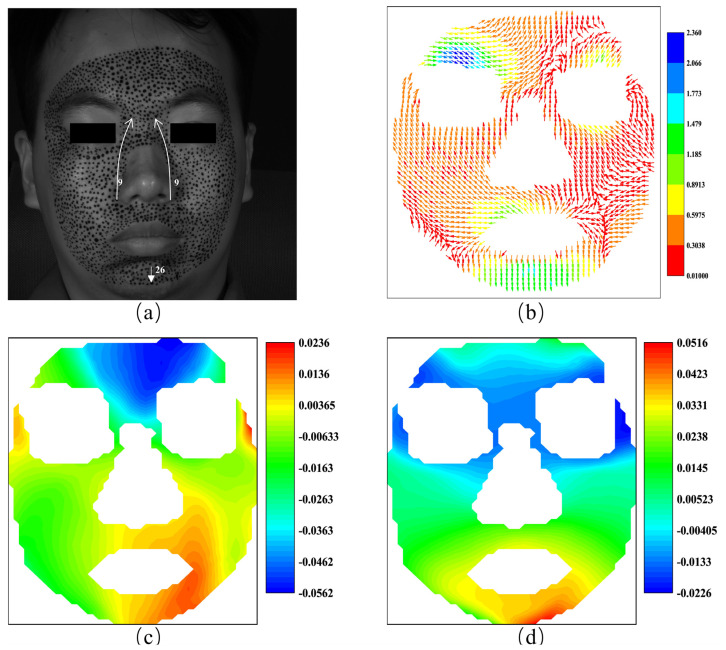
The AUs and the vector field and the strain fields at the expression of disgust: (**a**) the AUs caused by disgust; (**b**) the vector field of disgust; (**c**) horizontal strain field; (**d**) vertical strain field.

Figure 15 demonstrates the AU compositions of the classic FACS, the displacement vector and the horizontal and vertical strain fields of the subject under disgust. The AU 9 and AU 26 associated with disgust are shown in Figure 15a, indicating the nose wrinkles, the skin on the bridge lifts, and the nasal wings rise, enhancing the infraorbital triangle and deepening the upper nasolabial fold. This engagement of AUs reflects a complex interaction of facial muscles together to display disgust. Simultaneously, the upper lip elevates slightly, the jaw lowers to its fullest relaxed extent, and the lips part accordingly. As shown in Figure 15b, the displacement vectors exhibit an upward trend on both sides of the nostrils, corresponding to the movement represented by AU9. However, the downward displacement vectors at the chin perfectly correspond to AU26. And the magnitude of displacement vectors at the chin is greater than that at the sides of the nostrils.

The obvious negative horizontal strain appears on the forehead in Figure 15c, which is not directly reflected in the FACS but might indicate a potential muscle movement associated with the expression of anger. Meanwhile, Figure 15d shows the negative strain, i.e., the contraction at the location of the levator labii superioris alaeque nasi, and the positive strain, i.e., the tension at the location of the masseter. This appears to be caused by the contraction of the levator labii superioris alaeque nasi and the relaxation of the masseter. Additionally, the nuanced displacement and strain fields provide a deeper understanding of the muscular coordination involved in disgust. Specifically, the negative strain in the forehead, although not typical for disgust, might be the signal blending of emotional states or intensity of expression that involves broader muscle groups beyond the primary AUs of disgust.

**Figure 16:**
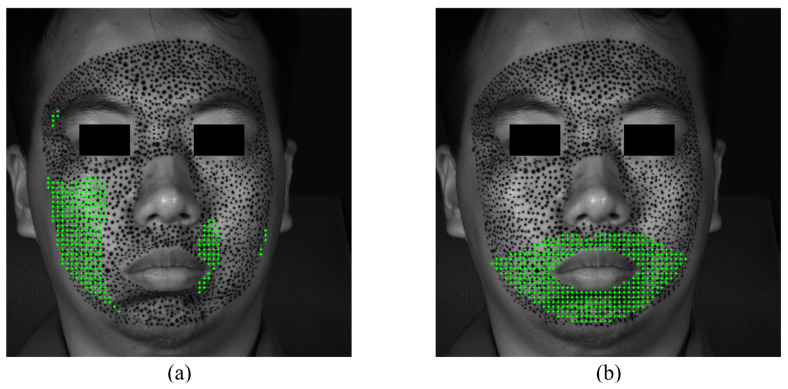
The S-POIs at the expression of disgust: (**a**) horizontal S-POIs; (**b**) vertical S-POIs.

The specific locations of S-POIs in the expression of disgust are shown in Figure 16a,b. The horizontal and vertical S-regions are observed to be larger than those in expressions of happiness or anger. The horizontal S-region, extending from beneath the eye to the jaw, suggests a significant involvement of the cheek muscles in disgust. The vertical S-region includes the area around the mouth, indicating that muscular activities related to disgust also prominently feature the muscles around the mouth. This extended distribution of S-POIs in the cheek and mouth areas implies a broad engagement of facial muscles, possibly reflecting the complex nature of disgust that involves a clear reaction from both the upper and lower face. The experimental data points to these regions as the initial zones of activation during the expression of disgust.

**Figure 17:**
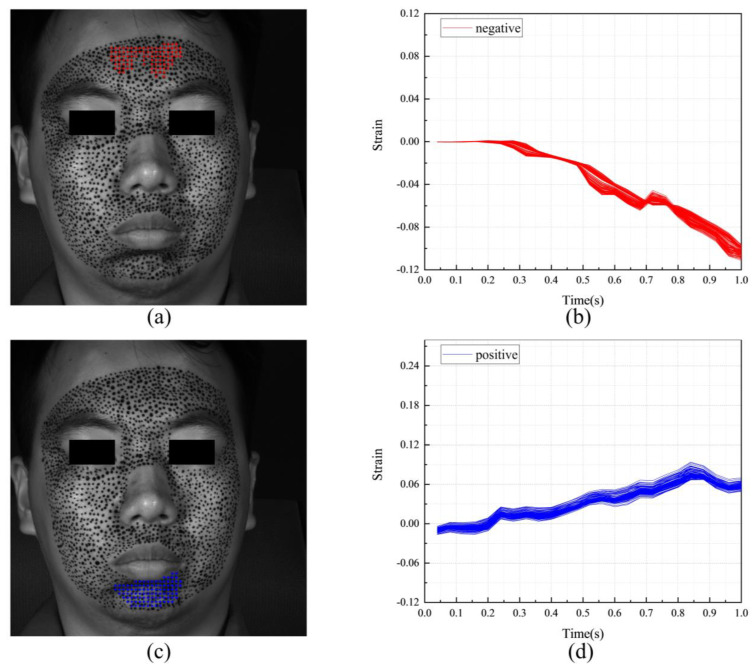
The F-region and the strain evolutions of F-POIs in the expression of disgust: (**a**) the horizontal F-region; (**b**) the horizontal strain evolutions of horizontal F-POIs; (**c**) the vertical F-region; (**d**) the vertical strain evolutions of vertical F-POIs.

Figure 17 demonstrates the measured F-region and the strain evolutions of F-POIs under disgust. The locations and the strain evolutions of the horizontal F-POIs are shown in Figure 17a and Figure 17b, respectively. The locations and the strain evolutions of the vertical F-POIs are shown in Figure 17c,d, respectively. The horizontal F-region is located in the middle of the forehead with negative strain, while the vertical F-region is found on the jaw with positive strain. This suggests that the most active areas in the expression of anger are located in the middle of the forehead and on the jaw. The strain fields reveal that the forehead and jaw exhibit opposite behaviors during disgust. The negative strain on the forehead indicates inward and downward movement, while the positive strain on the jaw reflects outward or expansive movement. This opposition suggests a complex muscular coordination that could be unique to the expression of disgust.

#### 4.3.4. Fear

**Figure 18:**
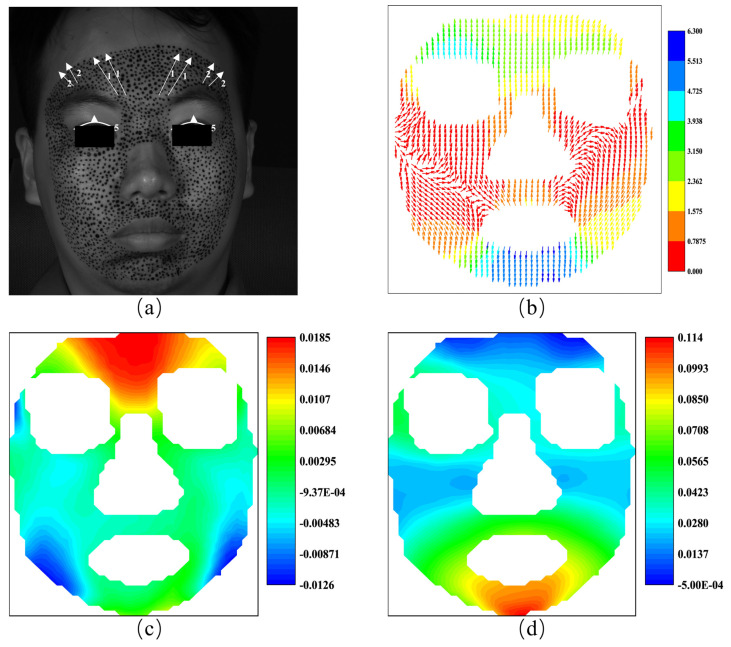
The AUs and the vector field and the strain fields at the expression of fear: (**a**) the AUs caused by fear; (**b**) the vector field of fear; (**c**) horizontal strain field; (**d**) vertical strain field.

The AU compositions of the classic FACS, the displacement vector, and the horizontal and vertical strain fields of the subject under fear are shown in Figure 18. Figure 18a displays AU 1, AU 2, and AU 5, resulting in the eyebrows being lifted, as indicated by the arrows, which reveals the eye cover fold and raises the upper eyelid to expose the iris fully. These actions are attributed to the contraction of the occipitofrontalis muscle. Similarly, in Figure 18b, the displacement vectors across the entire forehead predominantly point upwards, corresponding closely to the movements represented by AU1 and AU2, despite not showing a significant slant. And the displacement vectors show larger magnitudes at the upper left position of the eyebrow. The Figure 18b shows nearly the same movements and the contraction of the occipitofrontalis is responsible for such movements.

Figure 18c displays positive horizontal strain across the forehead, which indicates the state of tension, and Figure 18d shows negative vertical strain, which is indicative of contraction. These strain distributions are in direct correlation with the movements generated by AU 1 and AU 2. Moreover, the experiment also shows significant strain around the mouth area, although it is not traditionally associated with the fear response, as indicated by the classic FACS, suggesting a possible extension of the fear expression or co-occurrence of other emotional components.

**Figure 19:**
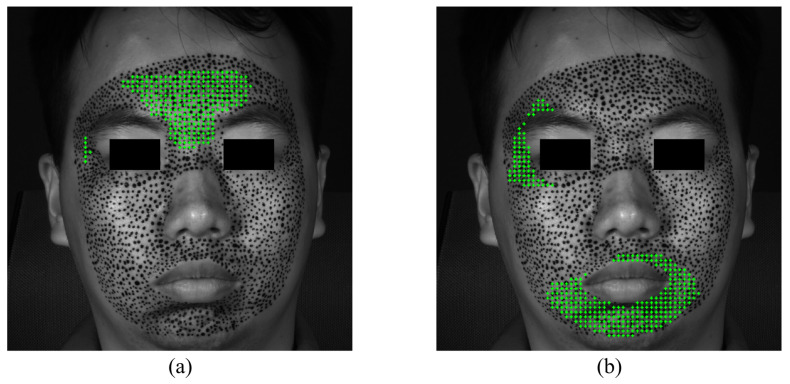
The S-POIs at the expression of fear: (**a**) horizontal S-POIs; (**d**) vertical S-POIs.

Figure 19 displays the locations of S-POIs for the expression of fear, with specific locations detailed in Figure 19a,b. The horizontal S-region is located on the forehead and the middle of the eyebrow, while the vertical S-region is primarily around the mouth and the canthus. Therefore, the experimental results indicate that the expression of fear originates from the forehead, middle of the eyebrow, areas around the canthus and the mouth. These S-regions suggest that fear expression is not only transmitted through eyebrow and forehead tension but also includes significant movement around the mouth and eyes in the initial stage, which indicates a more complex interplay of facial muscles than what may be traditionally associated with fear.

**Figure 20:**
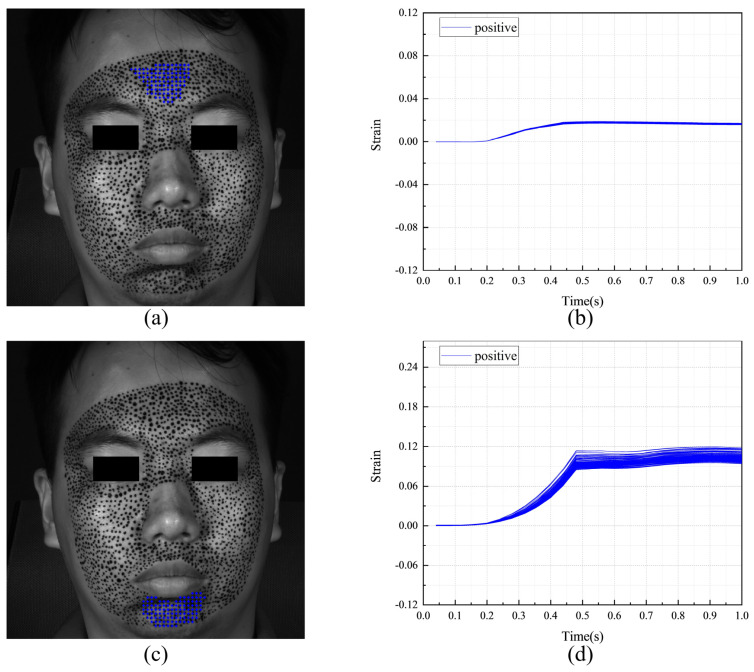
The F-region and the strain evolutions of F-POIs in the expression of fear: (**a**) the horizontal F-region; (**b**) the horizontal strain evolutions of horizontal F-POIs; (**c**) the vertical F-region; (**d**) the vertical strain evolutions of vertical F-POIs.

The F-region and the strain evolutions of F-POIs are showcased in Figure 20. The locations and strain evolutions of the horizontal F-POIs are detailed in Figure 20a,b, respectively. Similarly, the locations and strain evolutions of the vertical F-POIs are presented in Figure 20c,d, respectively. The horizontal F-region is situated in the middle of the forehead, and the vertical F-region is located in the middle of the jaw. Both the horizontal and vertical F-POIs exhibit positive strain. Therefore, during the formation of the expression of fear, the most significantly deformed regions are the middle of the forehead and the middle of the jaw. Figure 20b,d show that the strain evolutions of all F-POIs are closely aligned. At approximately 0.15 s, the strain of all F-POIs begins to increase and gradually disperse. By around 0.45 s, the strain of all F-POIs stabilizes. However, the vertical strain of the vertical F-POIs is larger and more dispersed than the horizontal strain of the horizontal F-POIs.

#### 4.3.5. Sadness

**Figure 21:**
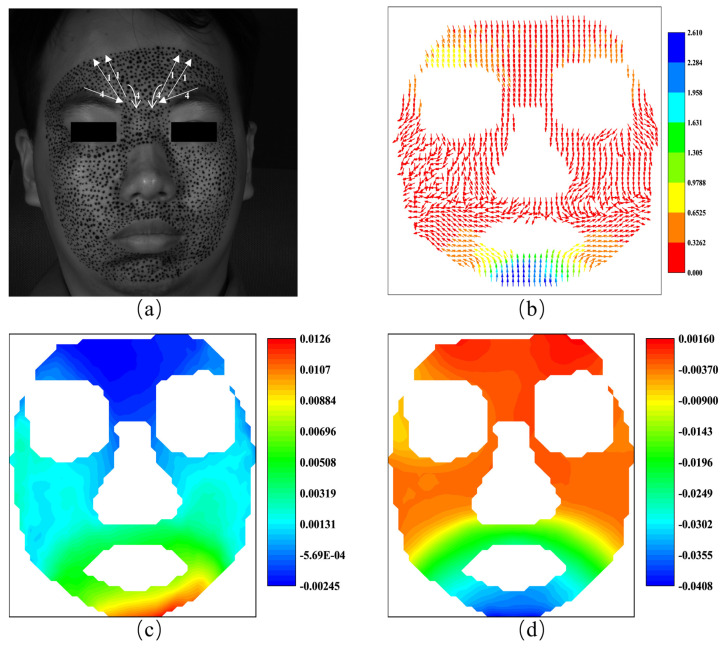
The AUs and the vector field and the strain fields at the expression of sadness: (**a**) the AUs caused by sadness; (**b**) the vector field of sadness; (**c**) horizontal strain field; (**d**) vertical strain field.

Figure 21 illustrates the AU compositions of the classic FACS, along with the displacement vector and the horizontal and vertical strain fields of the subject experiencing sadness. Figure 21a shows AU 1 and AU 4, indicating that the inner corners of the eyebrows are lifted and drawn together due to the contraction of the occipitofrontalis. The vector field depicted in Figure 21b may not clearly show the movement.

Nonetheless, Figure 21c,d reveal negative strain, i.e., contraction in the horizontal direction, and positive strain, i.e., tension in the vertical direction. These findings directly correspond to AU 1 and AU 4 and provide more detail. Notably, significant strain is observed at the jaw, which may be related to AU 15. The strain at the jaw suggests that the expression of sadness may involve a complex interaction of facial muscles, not limited to the upper face but including the lower face muscles as well, possibly reflecting a suppression of emotion or a more profound experience of sadness.

**Figure 22:**
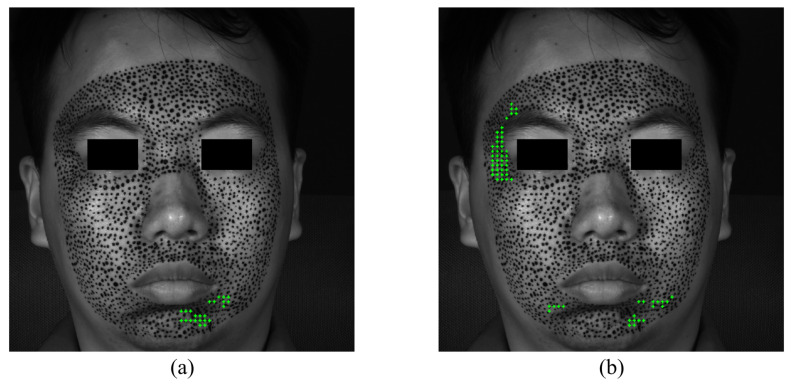
The S-POIs at the expression of sadness: (**a**) horizontal S-POIs; (**b**) vertical S-POIs.

The S-POIs for the expression of sadness are depicted in Figure 22. The horizontal S-region is a small area at the corner of the mouth, while the vertical S-region encompasses the canthus and the corner of the mouth. Experimental results indicate that the expression of sadness originates from the canthus and the corner of the mouth. Furthermore, the S-region associated with the expression of sadness is significantly smaller compared to other expressions. The concentration of S-POIs in these regions is less extensive but more focused compared to other expressions, possibly reflecting the inherent nature of the emotional state. The S-region associated with the expression of sadness is significantly smaller, indicative of a more localized involvement of facial muscles in the initial stage.

**Figure 23:**
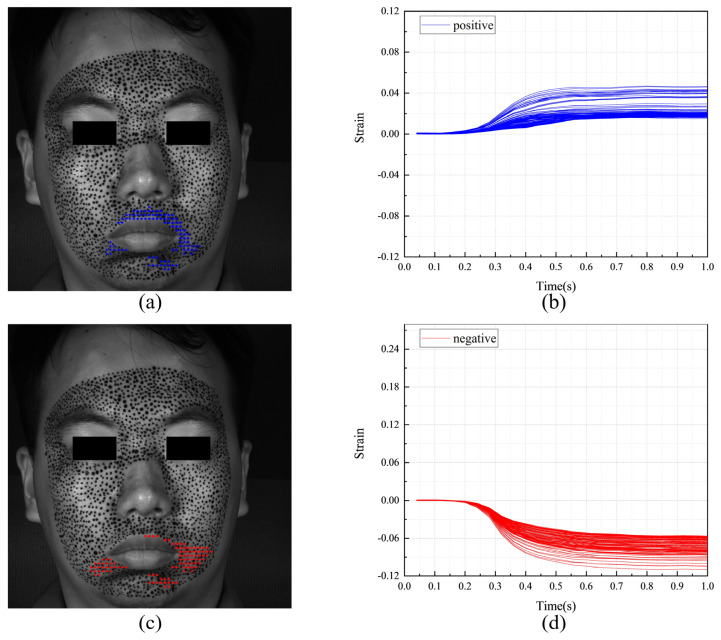
The F-region and the strain evolutions of F-POIs in the expression of sadness: (**a**) the horizontal F-region; (**b**) the horizontal strain evolutions of horizontal F-POIs; (**c**) the vertical F-region; (**d**) the vertical strain evolutions of vertical F-POIs.

Figure 23 displays the F-region and the strain evolutions of F-POIs, Figure 23a,b show the locations and strain evolutions of the horizontal F-POIs, and Figure 23c,d present the same for the vertical F-POIs. Both the horizontal and vertical F-regions are located around the mouth, with the horizontal F-POI strain remaining positive and the vertical F-POI strain staying negative. The areas of most activity and the trends in strain changes, whether horizontal or vertical, are generally consistent. Strain in all F-POIs begins to increase and disperse around 0.2 s and become stable by about 0.4 s. However, the vertical strain is noticeably larger than the horizontal strain. In addition, the amplitude of the vertical strain exceeds that of the horizontal strain, suggesting a greater degree of vertical stretching or compression in the facial movements associated with sadness.

#### 4.3.6. Surprise

**Figure 24:**
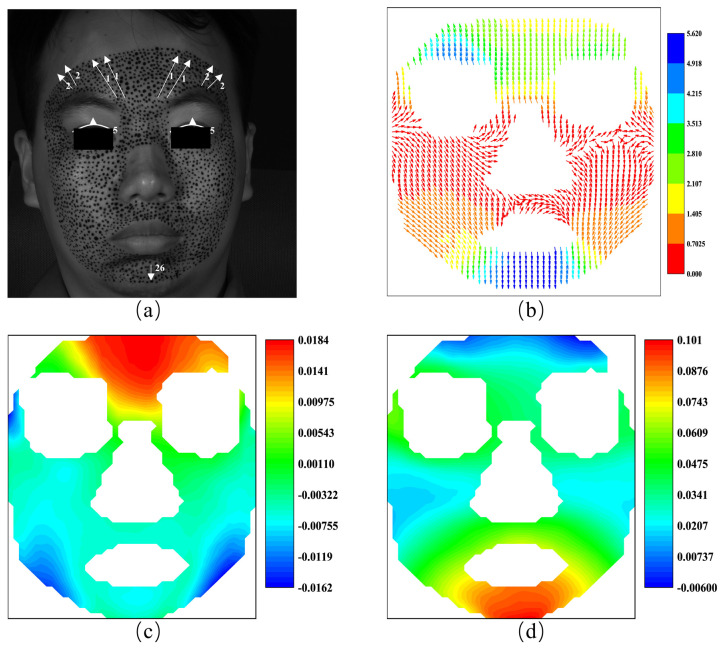
The AUs and the vector field and the strain fields at the expression of surprise: (**a**) the AUs caused by surprise; (**b**) the vector field of surprise; (**c**) horizontal strain field; (**d**) vertical strain field.

The AU compositions of the classic FACS, the displacement vector, and the horizontal and vertical strain fields of the subject under surprise are illustrated in Figure 24. Figure 24a displays AU 1 and AU 2, along with AU 5 and AU 26, where the contraction of the occipitofrontalis and the levator palpebrae superioris causes the eyebrows and upper eyelids to be raised. The lowering of the jaw and the parting of the lips are due to the relaxation of the masseter. From Figure 24b, the displacement vectors across the entire forehead predominantly point upwards, exhibiting varying magnitudes and angles. In particular, at the upper left position of the eyebrow, the displacement vectors show larger magnitudes and a slight leftward inclination, which is largely consistent with the movements represented by AU1 and AU2. Conversely, the displacement vectors at the chin point downwards and have significant magnitudes, corresponding to the movements represented by AU26. The vector field in Figure 24b suggests similar movements, with the movement of the jaw being significantly larger than in other areas.

From Figure 24c,d, the positive strain, i.e., tensions in the horizontal direction, and the negative strain, i.e., contraction in the vertical direction on the forehead, imply the status of the occipitofrontalis clearly. Additionally, significant strain is observed on the two sides of the face in the horizontal direction, which suggests a considerable relaxation of the masseter muscle during the expression of surprise. This relaxation likely corresponds to the lowering of the jaw and the separation of the lips, which are integral components of the facial configuration of surprise. Furthermore, this strain distribution may also indicate the coordinated action of facial muscles in expressing the expression, particularly reflecting dynamic changes between the upper and lower jaw.

**Figure 25:**
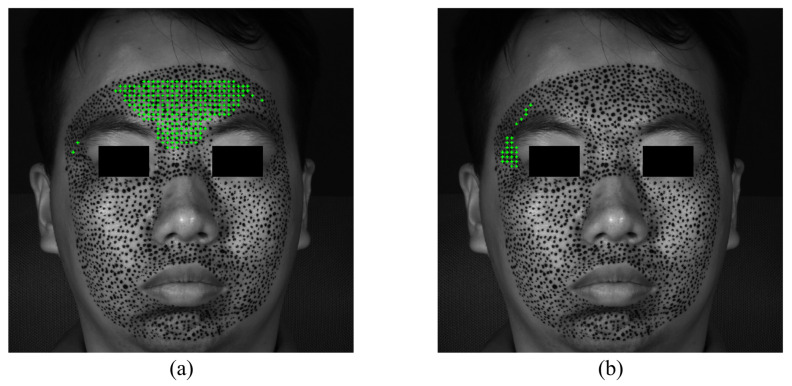
The S-POIs at the expression of surprise: (**a**) horizontal S-POIs; (**b**) vertical S-POIs.

The S-region associated with the expression of surprise is depicted in Figure 25. The horizontal S-region is primarily located on the forehead and in the middle of the eyebrows, while the vertical S-region is considerably smaller, confined to a small area at the corner of the eye. Experimental results indicate that the expression of surprise primarily originates from the forehead and the middle of the eyebrows. The location of the vertical S-POIs at the eye corners might reflect the subtle rapid movements that occur around the eye when one is startled.

**Figure 26:**
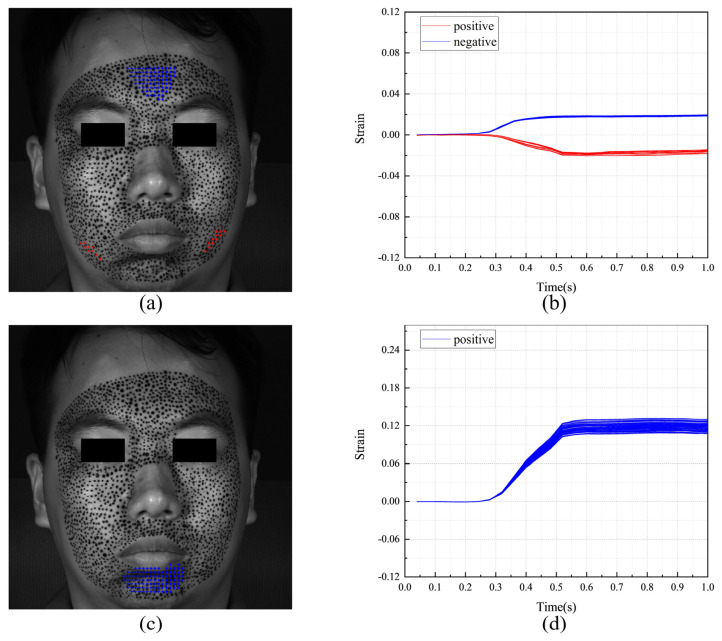
The F-region and the strain evolutions of F-POIs in the expression of surprise: (**a**) the horizontal F-region; (**b**) the horizontal strain evolutions of horizontal F-POIs; (**c**) the vertical F-region; (**d**) the vertical strain evolutions of vertical F-POIs.

Figure 26 illustrates the F-region and the strain evolutions of F-POIs. The locations and strain evolutions of the horizontal F-POIs are depicted in Figure 26a,b, respectively. Similarly, the locations and strain evolutions of the vertical F-POIs are shown in Figure 26c,d. The horizontal F-region is split into two parts: one in the middle of the forehead with positive strain, and the other on both sides of the lower jaw with negative strain. The vertical F-region, located in the middle of the jaw, exhibits positive strain. The most active areas in the horizontal direction, the middle of the forehead and the lower jaw, have nearly identical strain magnitudes but opposite directions. The area with the most severe deformation is the middle of the jaw, where the strain magnitude is slightly less than that of other F-POIs. Nonetheless, the trends of all F-POIs are broadly consistent, with strain beginning to increase and disperse around 0.3 s and becoming stable by approximately 0.5 s. These findings suggest that the surprise expression is affected by different muscles in the face working together, with the forehead and jaw acting together but in different ways to create a clear facial expression. The strong change in the middle of the jaw could be a key part of showing surprise, involving movements in the lower face, as well as lifting the brows.

#### 4.3.7. Comparison of the Different Expressions

**Figure 27:**
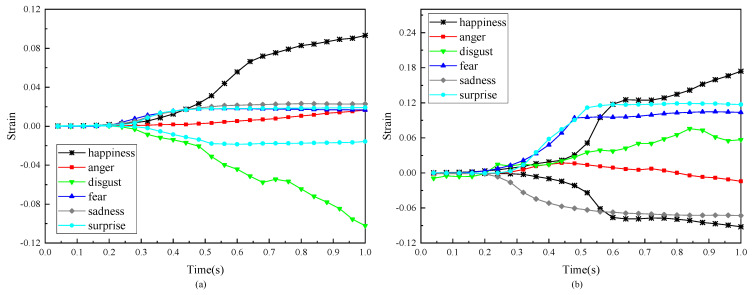
The evolutions of the mean value of the strain of F-POIs in 6 basic expressions: (**a**) the evolutions of the mean value of horizontal strain; (**b**) the evolutions of the mean value of vertical strain.

Since the horizontal and vertical strain evolutions of F-POIs in each expression present similar shapes with different magnitudes, the mean strain evolutions of all expressions are calculated. Figure 27 shows the results so that we can identify the characteristics of the strain data when the subject is under different expressions. Due to the above datum being from merely one subject, the following discussion on the different expressions is limited to this subject in this experiment and does not have universality. In the future, if we can conduct DIC-based measurements across a large number of subjects, it can provide us with a substantial volume of quantitative data for objective facial expression recognition.

Figure 27a,b show the results on the horizontal and vertical strains, respectively. First of all, the stain magnitudes of different expressions are significantly different. For example, in Figure 27a,b, it is happiness that has the most significant strain. Furthermore, it should be noticed that, with respect to the horizontal stain in Figure 27a, happiness, anger, fear and sadness display tension in their F-region, while disgust shows contraction. Additionally, the evolutions of vertical strain are more intense and more distinctive. If we are concerned with the time evolutions of all expressions, it is indicated that the formation processes of all expressions take less than 1 s.

### 4.4. Discuss

DIC has been widely applied in the fields of engineering and science. Generally, the acceptable accuracy for displacement in DIC ranges from 0.1 to 0.05 pixels, while the accuracy for strain is around 0.0001. The accuracy of DIC measurements is influenced by various factors, including the choice of camera, interpolation functions, and shape functions [28]. The hardware and algorithms applied in this study are commonly utilized in DIC applications, offering relatively high performance. Most importantly, as the most crucial factor affecting DIC accuracy, the speckle pattern brings some inconveniences and limitations in the experimental process. But, it has been effectively utilized in this study.

On the other side, this study places a particular emphasis on the role of strain in analyzing facial expressions. However, given that the experimental subject is a living human, inevitable physiological activities such as breathing or rigid facial motion occur, potentially introducing calculation errors. Consequently, the preliminary experiments detailed in Section 4.2 have established a strain threshold of 0.002 to account for these variables. Based on the experiments detailed in Section 4.3, the strain values of expressions range from −0.1 to 0.18, significantly exceeding the threshold that accounts for calculation errors. Hence, the measurement results of this study are considered to be reliable and trustworthy.

In terms of the time required for measurement, the computation time required for DIC is determined by various factors. In standard computations, the selection of shape functions and interpolation methods, as well as the quantity of POIs, will all influence the computation time. Moreover, the performance of the computer can also impact computation time if parallel processing is employed using CPUs or GPUs. In this work, due to our implementation of the self-adaptive reference image selection strategy, the rate and magnitude of facial deformation affect the choice of reference images during computation, thus affecting computation time to a certain degree. In order to ensure computational accuracy, the computations in this paper are conducted offline rather than in real time. Based on the experimental results, facial expressions are generally formed within approximately 0.7 s. We utilized a time resolution of 0.04 s to capture images well beyond the 0.7 s duration for computation. Although this significantly increased the computational load and processing time, it ensured the observation of more dynamic details.

## 5. Conclusions

In this paper, 3D-DIC, a classical deformation measurement technique developed in the field of experimental mechanics, is adapted to analyze the facial deformation when the subject is experiencing the six human basic expressions. Moreover, to make the traditional 3D-DIC more appropriate for measuring the dynamic deformation of facial expressions, some customized improvements are proposed to make the measurements with high resolution, low noise level and acceptable computing loading. An inherent merit of 3D-DIC is its ability to provide the displacement and strain fields of 3D deformation. These rich data on facial deformation can facilize quantitative expression analysis. Due to its locale nature, the strain fields can display an intuitive reflection of tension or contraction of the relevant muscles. Furthermore, the horizontal and vertical strains separately supply us with different details on the facial expression. Based on the captured deformation evolution, two novel concepts, S-region and F-region, are proposed to characterize the dynamic process of different expressions. The features of S-region and F-region under different expressions are discussed in detail. This work provides a systematic and generalizable analysis method, and the application of this method could obtain more representative datasets. Such datasets are expected to offer strong quantitative support to further reveal more potential diversity and regularity in facial expressions. Moreover, 3D-DIC heavily relies on the speckle patterns on the surface, which introduces some limitations and tedious processes. Reducing the dependence on speckle patterns in 3D-DIC is of great significance in generalizing the method for analyzing expressions.

## Data Availability

Data are contained within the article.
